# *Brucella abortus* Infection Modulates 3T3-L1 Adipocyte Inflammatory Response and Inhibits Adipogenesis

**DOI:** 10.3389/fendo.2020.585923

**Published:** 2020-09-18

**Authors:** Ayelén Ivana Pesce Viglietti, Guillermo Hernán Giambartolomei, Jorge Quarleri, María Victoria Delpino

**Affiliations:** ^1^ Instituto de Inmunología, Genética y Metabolismo (INIGEM), Facultad de Farmacia y Bioquímica, Universidad de Buenos Aires (UBA), Consejo Nacional de Investigaciones Científicas y Técnicas (CONICET), Buenos Aires, Argentina; ^2^ Instituto de Investigaciones Biomédicas en Retrovirus y Sida (INBIRS), Facultad de Medicina, Universidad de Buenos Aires (UBA), Consejo Nacional de Investigaciones Científicas y Técnicas (CONICET), Buenos Aires, Argentina

**Keywords:** Brucella, TNF-α, adipogenesis, macrophage, inflammation

## Abstract

Brucellosis is a prevalent global zoonotic infection but has far more impact in developing countries. The adipocytes are the most abundant cell type of adipose tissue and their secreted factors play an important role in several aspects of the innate and adaptive immune response. Here, we demonstrated the ability of *Brucella abortus* to infect and replicate in both adipocytes and its precursor cells (pre-adipocytes) derived from 3T3-L1 cell line. Additionally, infection of pre-adipocytes also inhibited adipogenesis in a mechanism independent of bacterial viability and dependent on lipidated outer membrane protein (L-Omp19). *B. abortus* infection was able to modulate the secretion of IL-6 and the matrix metalloproteases (MMPs) -2 and-9 in pre-adipocytes and adipocytes, and also modulated de transcription of adiponectin, leptin, and resistin in differentiated adipocytes. *B. abortus*-infected macrophages also modulate adipocyte differentiation involving a TNF-α dependent mechanism, thus suggesting a plausible interplay between *B. abortus*, adipocytes, and macrophages. In conclusion, *B. abortus* is able to alter adipogenesis process in adipocytes and its precursors directly after their infection, or merely their exposure to the *B. abortus* lipoproteins, and indirectly through soluble factors released by *B. abortus*-infected macrophages.

## Introduction

Brucellosis is a prevalent global zoonotic infection but has far more impact in developing countries (1). Clinical manifestations of brucellosis are frequently associated with inflammatory responses in the organs affected (2). Adipose tissue represents one of the largest organs and constitutes up to 25% of the mass of the body in normal weight individuals (3). It is distributed throughout the body and is made up of different cell types that are involved in storing energy, regulating metabolism in addition to fulfilling neuroendocrine and immunological functions (3). Adipocytes secrete a multiplicy of adipokines, including adiponectin, leptin, and various cytokines such as IL-1β and IL-6. The resident immune cells—which include lymphocytes and macrophages—also secrete multiple inflammatory mediators, again including classical cytokines and chemokines (4, 5).

Adipocytes are the most abundant cell type of adipose tissue and their secreted factors play an important role in several aspects of the innate and adaptive immune response among other functions (6). Therefore, these cells could participate in the modulation of the immune response during *Brucella* infection. On the other hand, adipocytes are specialized for fat storage regulating the balance of energy and homeostasis (7). The pathogens could take advantage of this characteristic, turning adipose tissue into a survival niche. In fact, *Mycobacterium tuberculosis*, *Trypanosoma cruzi*, influenza virus A, *Chlamydia pneumoniae*, HIV -among other pathogens- are housed in adipose tissue (8–11). The cell and tissue in which *Brucella* persists during chronic infection remain largely unknown. Most studies describe the location of the bacteria based on the site of isolation or the histopathology of the disease, but the place where the bacterium resides has not been elucidated (12, 13).

Adipocytes differentiate from mesenchymal stem cells. During the differentiation process, the transcriptional factor CCAAT/enhancer-binding protein (C/EBP)β is transiently induced leading to activation of two master adipogenic transcription factors, peroxisome proliferator-activated receptor (PPAR)γ, and C/EBPα. These mediators stimulate each other to activate the transcription of genes implicated in lipid metabolism (14). Adipocyte differentiation is regulated by hormones, cytokines, growth factors, and also by matrix metalloproteinase (MMPs) (15, 16). A recent study performed in canine fetuses and neonates naturally infected with *B. canis* revealed its intracellular localization in adipocytes. *B. canis* was localized near to the lipid droplet and in the same place of the endoplasmic reticulum. The adipocytes from neonates and fetuses are immature and present features of pre-adipocytes. During their differentiation process, the pre-adipocytes express unfolded proteins as occurs during the intracellular replication of *Brucella* spp. (17, 18). This finding suggests that pre-adipocytes could be a replicative niche for *Brucella* spp. during the differentiation process. In the present study, we investigate whether *B. abortus* can infect and survive in differentiated adipocyte and its precursors, as well as the incumbency on both adipogenesis and immune response modulation, on the inflammatory response during brucellosis.

## Materials and Methods

### Bacterial Culture

*Brucella abortus* S2308, DsRed-expressing *B. abortus* 2308 (provided by Diego Comerci, UNSAM University, Argentina), were grown for 18 h in 10 ml tryptic soy agar supplemented with yeast extract (Merck) with constant agitation (150 rpm) at 37°C. Bacteria were collected and inoculums prepared as previously described (19). To obtain heat-killed *B. abortus* (HKBA), bacteria were washed with sterile physiological solution and heat-killed at 70°C for 20 min. Absence of bacterial viability was verified by the lack of its *in vitro* growth on trypticase soy agar (TSA). Live *B. abortus* manipulations were performed in biosafety level 3 facilities.

### Cell Culture

3T3-L1 fibroblasts were obtained from the American Type Culture Collection (ATCC, Manassas, VA) and were culture in DMEM (Gibco) containing 10% of heat-inactivated fetal bovine serum (FBS) (Gibco), 2 mM of L-glutamine (Gibco), 1 mM of sodium pyruvate (Gibco), and penicillin-streptomycin. The murine macrophage cell line J774 was grown in DMEM with 10% FBS and supplemented as previously described.

### Adipocyte Differentiation

3T3-L1 cells were seeded at 5 x 10^4^ cells/well in 24-well plates and allowed to reach confluence. After 2 days (day 0), the medium was changed to differentiation medium (DMEM, 0.5 mM 3-isobutyl-1-methylxanthine (IBMX), 1 µM dexamethasone (DM), and 1 µg/ml human insulin), all from SIGMA. At day 2, the medium was replaced by a maintenance medium (10% FBS and 1 µg/ml insulin). Full differentiation was reached at day 10–15.

Adipocyte differentiation was evaluated by oil red O staining (Sigma). Cultures in 24-well plates were fixed for 1 h with 10% formalin and then washed with 60% isopropanol, stained for 30 min by complete immersion in a working solution of 6% oil red O, and wash repeatedly with water. Ten microscopic fields per well in three wells per condition were quantified for each experiment. The percentage of adipocytes was calculated for the nontreated/noninfected controls.

### Cellular Infection

3T3-L1 preadipocytes and adipocytes were separately seeded in two different densities: 2x10^4^ cells per well in 24 well plate, and 1x10^5^ cell per well in 6 well plate. Besides, J774.A1 macrophages were seeded at a density of 3x10^5^ cells per well in 24 well plates. These three cell types were infected with *B. abortus* S2308 or DsRed-expressing *B. abortus* S2308 at different multiplicities of infection (MOI) 100 to 1,000 or at MOI 100 for J774.A1 cell line. After the bacterial suspension was dispensed, the plates were centrifuged for 10 min at 2,000 rpm and then incubated for 2 h at 37°C under a 5% CO2 atmosphere. Cells were extensively washed with DMEM to remove extracellular bacteria and incubated in medium supplemented with 100 μg/ml gentamicin and 50 μg/ml streptomycin to kill extracellular bacteria. Adipocytes and preadipocytes were harvested at different times to determine cytokine production, MMP secretion, lipid droplets staining and adiponectin, leptin, PPAR-γ, C/EBP-α, C/EBP-β gene transcription. Supernatants from J774.A1 macrophages were harvested at 24 h post-infection and sterilized by filtration through a 0.22 nitrocellulose filter. To evaluate the intracellular replication of *B. abortus*, the infected cells were washed and lysed at different time post-infection with 0.2% (vol/vol) of triton X-100. The number of viable intracellular bacteria was determined by the count of CFU/ml from serial dilutions to the tenth in TSA plates.

### *Brucella*-Derived Lipoproteins and LPS

*B. abortus* L-Omp19 and U-Omp19 were obtained as previously described (20). Both recombinant proteins contained 0.25 endotoxin U/µg protein, as assessed by Limulus amoebocyte lysates (Associates of Cape Cod, East Falmouth, MA, USA). Protein concentration was determined by the BCA method (Pierce, Rockford, IL, USA). Ignacio Moriyon from the University of Navarra, Pamplona, Spain kindly provided *B. abortus* S2308 LPS and *E. coli* O111K58H2 LPS. Pam_3_Cys was acquired from Boehringer Mannheim (Indianapolis, IN, USA).

### Confocal Microscopy

3T3-L1 differentiated cells seeded onto glass coverslips were infected with DsRed expressing-*B. abortus* S2308 at MOI 100 as was previously described. At different time points, cells were fixed with paraformaldehyde, permeabilized with 0.3% Triton X-100, and then lipid droplets were stained with 1 µg/ml of Bodipy 493/503 (Invitrogen), and nucleus were stained with TO-PRO^®^-3 (Invitrogen). The coverslips were mounted in PBS-glycerin (9:1 vol/vol) and analyzed in a FV-1000 confocal microscope with an oil-immersion Plan Apochromatic 60X NA1.42 objective (Olympus). Ten microscopic fields per well in 3 wells per condition were quantified for each experiment. The percentage of adipocytes was calculated to the noninfected controls. To determine *B. abortus* replication we visualized DsRed-expressing *B. abortus* positive areas by confocal microscopy and quantified using Image J software (National Institutes of Health).

The amount and individual diameter size of the lipid droplets in the image were measured using Image J software and data were loaded into GraphPad Prism 5.0 (GraphPad Software, La Jolla, CA, USA) and evaluated for average lipid droplet size and size-frequency distribution for individual adipocytes. The adipocytes containing lipid droplets with a mean diameter >1 µM were classified as cells with big lipid droplets size.

### Measurement of Cytokine Concentrations

Secretion of IL-1β, IL-6, and TNF-α were quantified by ELISA in culture supernatants following the manufacturer’s instructions (BD Pharmingen, San Diego, CA).

No cross-reactivity with other mouse cytokines was identified. For IL1β ELISA were tested IL-1β IL-2, IL-3, IL-4, IL-5, IL-6, IL-7, IL-9, IL-10, IL-12 (p70), IL-15, IFN-γ, MCP-1, TCA3, and TNF-α; for IL-6 ELISA were tested IL-1β, IL-2, IL-3, IL-4, IL-5, IL-7, IL-9, IL-10, IL-12 (p70), IL-15, IFN-γ, GM-CSF, MCP-1, TCA3, and TNF-α; for IL1 β ELISA were tested IL-2, IL-3, IL-4, IL-5, IL-6, IL-7, IL-9, IL-10, IL-12 (p70), IL-15, IFN-γ, MCP-1, TCA3, and TNF-α. Sensitivity for IL-1 β, IL-6, and TNF-α the limit of detection is 15.6 pg/ml.

### Zymography

Gelatinase activity was assayed by the method of Hibbs et al. with modifications, as described (21, 22). Briefly, a total of 20 μl of cell culture supernatants from infected cells or untreated controls were mixed with 5 μl of 5X loading buffer [0.25 M Tris (pH 6.8), 50% glycerol, 5% SDS, and bromophenol blue crystals] and loaded onto 10% SDS-PAGE gels containing 1 mg/ml gelatin (Sigma-Aldrich, Buenos Aires, Argentina). Following electrophoresis, gels were washed with a solution containing 50 mM Tris-HCl (pH 7.5) and 2.5% Triton X-100 (buffer A) for 30 min and with buffer A added with 5 mM CaCl2 and 1 μM ZnCl2 for 30 min and were later incubated with buffer A with additional 10 mM CaCl2 and 200 mM NaCl for 48 h at 37°C. Gelatin activity was visualized by the staining of the gels with 0.5% Coomassie blue. Unstained bands indicated the presence of gelatinase activity.

### Gelatinase Activity Under Native Conditions

Gelatinase activity in unprocessed culture supernatants (native conditions) was measured by using a gelatinase/collagenase ﬂuorometric assay kit (EnzChek; Invitrogen, Carlsbad, CA) according to the manufacturer’s instructions. The EnzChek kit contains DQ gelatin, a ﬂuorescein-conjugated gelatin so heavily labeled with ﬂuorescein that ﬂuorescence is quenched. When this substrate is digested by gelatinases or collagenases it yields highly ﬂuorescent peptides, and ﬂuorescence increase is proportional to proteolytic activity. Collagenase puriﬁed from *Clostridium histolyticum* provided in the assay kit serves as a control enzyme. Plates were read in a ﬂuorescence plate reader (Victor3; Perkin-Elmer, Waltham, MA).

### mRNA Extraction and Quantitative Real-Time PCR

Total RNA was extracted from cells using the kit Quick-RNA MiniPrep Kit (Zymo Research) according to the manufacturer’s instructions. cDNA was synthesized from 1 μg total RNA with the enzyme reverse transcriptase Improm-II (Promega). Real-time PCR was done with a SYBR green as a DNA binding fluorescent dye using a StepOne Real-Time PCR System (Applied Biosystems). The pairs of primers used were the following: adiponectin sense:5´**-**GACGACACCAAAAGGGCTCA-3´, antisense: 5´-GAGTGCCATCTCTGCCATCA-3´, leptin sense: 5´-TCCCTGCCTCAGACCAGTG-3´, antisense: 5´-TAGAGTGAGGCTTCCAGGACG-3´, PPAR-γ sense: 5´-CTGATGGCATTGTGAGACAT-3´, antisense: 5´-ATGTCTCACAATGCCATCAG-3´, C/EBP-α sense: 5´-TGTGCGAGCACGAGACGTC-3´, antisense: 5´-AACTCGTCGTTGAAGGCGG-3´, C/EBP-β sense: 5´-GCTGAGCGACGAGTACAAGA-3´, antisense: 5´-CAGCTCCAGCACCTTGTG-3´, β-actin sense: 5´-AACAGTCCGCCTAGAAGCAC-3´, antisense: 5´-CGTTGACATCCGTAAAGACC-3´.

The amplification cycle for adiponectin, leptin, PPAR-γ, C/EBP-α, and C/EBP-β was the following 10 min 95 °C, 40 cycles for 15 s at 95°C, 60°C for 30 s, and 72°C for 60 s. All primer sets yielded a single product of the correct size. The fold change (relative expression) in gene expression was calculated using the relative quantitation method (2^−ΔΔCt^). Relative expression levels were normalized against β-actin. Intra experiment CT values differences between samples were less than 0.5.

### Statistical Analysis

Each experiment was performed at least three times with different culture preparations. Data were represented as mean ± SD measured in triplicate from at least three individual experiments. Statistical analysis was performed with one-way ANOVA. Multiple comparisons between all pairs of groups were made with Tukey’s posttest, and those against two groups were made with Student᾽s *t* test, Mann-Whitney test. To determine normality, the Shapiro-Wilk normality test was used. Graphical and statistical analyses were performed with GraphPad Prism 5.0 software. P<0.05 was the minimum level for accepting a statistically significant difference between groups.

## Results

### *B. abortus* Infects and Replicates in Both Pre-Adipocytes and Adipocytes, and Inhibits Adipogenesis

**Figure 1:**
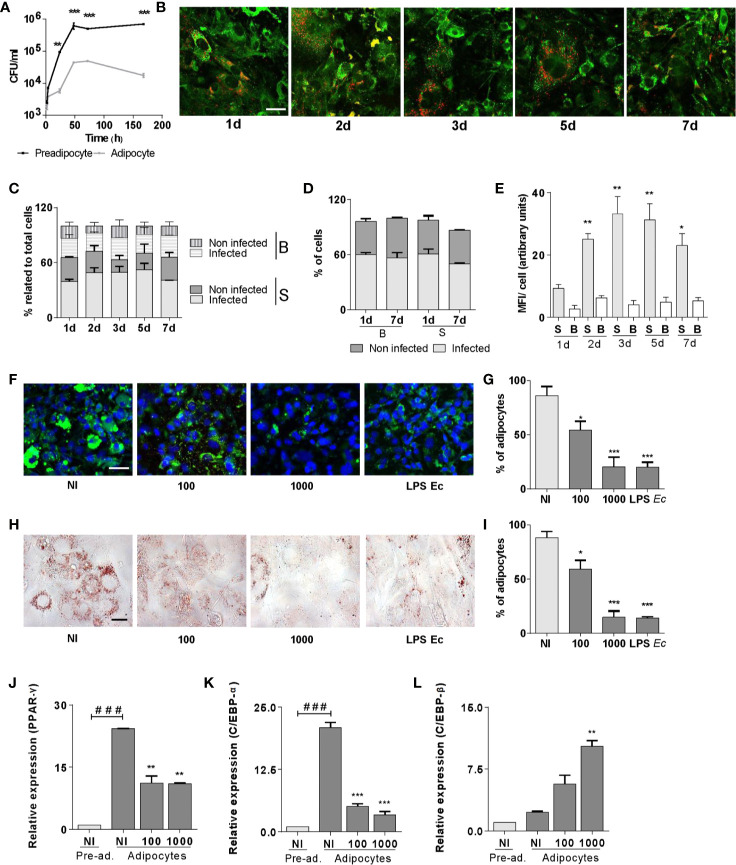
*B. abortus* replicates in pre-adipocytes and adipocytes and inhibits adipogenesis. Infection with *B. abortus* was performed at a multiplicity of infection (MOI)= 100, and CFU was determined at different times of infection in pre-adipocytes and adipocytes. **(A)**. Intracellular DsRed-expressing *B. abortus* in adipocytes assessed by confocal microscopy, lipid droplets were stained with Bodipy 493/503 **(B)**. Quantification of the experiment performed in B **(C–E)**. Percentage of infected and non-infected adipocytes with small (S) and big **(B)** lipids droplets respect to total cells at each time **(C)**. Percentage of infected and non-infected cells at 1 and 7 d post infection present in adipocytes with S or B droplets cells respect to total S o B cells at each time. Quantification of bacterial replication is measured as means of fluorescence intensity (MFI) **(E)**. Effect of *B. abortus* infection at MOI 100 and 1,000 on adipocyte differentiation determined by lipid droplets staining with Bodipy 493/503 **(F)**, quantified by cell counts **(G)** and Oil Red O staining **(H)**, quantified by cell counts **(I)**. Effect of *B. abortus* infection on PPAR-γ **(J)**, C/EBP-α **(K)**, and C/EBP-β **(L)** transcription determined by RT-qPCR in adipocytes after 24 h post-infection. LPS from *E. coli* (LPS *Ec*) was used as a positive control. Scale bar: 30 µm. Data are given as the mean ± SD measured in triplicate from at least three individual experiments. Ten microscopic fields per condition were quantified for each experiment. Data shown are from a representative experiment of three performed. *P < 0.05; **P < 0.01; ***P < 0.001vs non infected cells (NI). ^###^P< 0.001 vs non infected pre-adipocytes (NI/Pre-ad.).

There are no former reports about the interaction between *B. abortus* and adipogenic cells. Thus, we first determined the differential capacity of *B. abortus* to infect pre-adipocytes and adipocytes. As shown in **Figures 1A**. *B. abortus* was internalized by both, pre-adipocytes and adipocytes *in vitro*. However, the multiplication efficiency was dissimilar between them. In pre-adipocytes the number of intracellular bacteria was increased by more than one log at 24 h post-infection and continued growing thereafter. By contrast, adipocytes were less permissive for *B. abortus* growth, and the number of bacteria in adipocytes was significantly lower than that observed for pre-adipocytes (p < 0.01). The number of intracellular bacteria in adipocytes increased by one log at 48 h post-infection and then decline.

At seven days of adipogenic differentiation, the culture is heterogeneous with cells that present small and big lipid droplets (more than 1 µm in diameter). At this time, cells were infected with DsRed-expressing *B. abortus* and lipid droplets were stained with Bodipy 493/503, and cells were evaluated at different times post-infection. Our results indicate that *B. abortus* invades adipocytes in a manner that was independent of the lipid droplets size (**Figures 1B–D**). However, *B. abortus* preferentially replicates in adipocytes with small lipid droplets (**Figure 1E**).

To assess whether the infection affects adipocyte differentiation, pre-adipocytes were infected with *B. abortus* at MOI 100 and 1,000, in the presence of differentiation medium for 2 days, and then incubated with maintenance medium. *E. coli* LPS was used as a control. At 15 days post-infection cells were fixed. The presence of differentiated adipocytes was revealed by lipid droplets staining with Bodipy 493/503 and Oil Red O (**Figures 1F–I**). *B. abortus* infection inhibited adipocyte differentiation as was revealed by a reduction of lipid droplets formation.

Given the ability of *B. abortus* infection to inhibit adipocyte differentiation, subsequent experiments were carried out to determine whether such infection could also modulate the transcription of the essential pro-adipogenic factors C/EBP-α, C/EBP-β, and PPAR-γ (14). For this purpose, pre-adipocytes were infected, incubated with a differentiation/maintenance medium, and mRNA levels of mentioned pro-adipogenic factors were measured at 15 days. *B. abortus* infection promoted a decrease in the transcription of C/EBP-α and PPAR-γ genes despite an increase in the transcription of C/EBP-β gen (**Figures 1J–L**). Altogether, these results indicate that *B. abortus* replicates preferentially in pre-adipocytes slowing down their normal adipogenesis.

### *B. abortus* Infection Modulates Proinflammatory Cytokines and Adipokines Secretion in Pre-Adipocytes and Adipocytes

**Figure 2:**
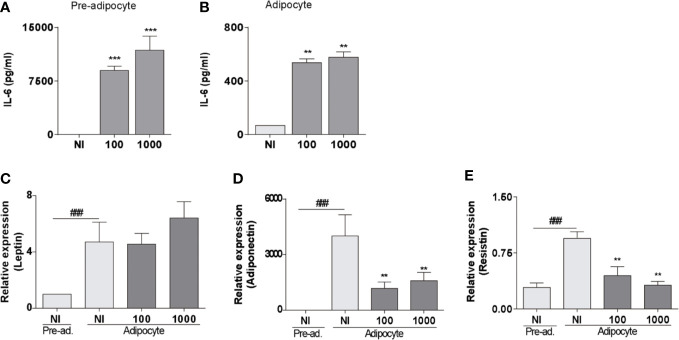
*B. abortus* infection modulates proinflammatory cytokines and adipokines secretion in pre-adipocytes and adipocytes. ELISA determination of IL-6 in pre-adipocytes **(A)** and adipocytes **(B)** measured in culture supernatants at 24 h post-infection. Determination of leptin **(C)**, adiponectin **(D)**, and resistin **(E)** by RT-qPCR in *B. abortus* -infected adipocyte cells at MOI 100 and 1,000 at 24 h post-infection. Data are given as the mean ± SD measured in triplicate from five individual experiments. Data shown are from a representative experiment of five performed **P < 0.01; ***P < 0.001vs non infected cells (NI). ^###^P< 0.001 vs non infected pre-adipocytes (NI/Pre-ad.)

The adipose tissue physiology is committed to the expression of cytokines and adipokines. They not only control fat metabolism but also immune homeostasis (6). Infection with *B. abortus* at MOI 100 and 1,000 induced IL-6 (**Figures 2A, B**) but not IL-1β nor TNF-α (not shown) secretion by both pre-adipocytes and adipocytes. In adipocytes, the mRNA transcription level of leptin remained unaltered after the *B. abortus* infection, but the levels of mRNA of adiponectin and resistin are significantly (p < 0.01) inhibited respect to uninfected control cells (**Figures 2C–E**). This is in concordance with the inhibitory effect of *B. abortus* infection on adipogenesis, since transcription of adipokine genes are controlled by the transcription factor PPAR-γ (23). These results indicate that *B. abortus* infection modulates the expression of cytokines and adipokines in pre-adipocytes and adipocytes.

### *B. abortus* Infection Induces MMPs Secretion in Pre-Adipocytes but Was Unable to Modulate MMPs in Adipocytes

**Figure 3:**
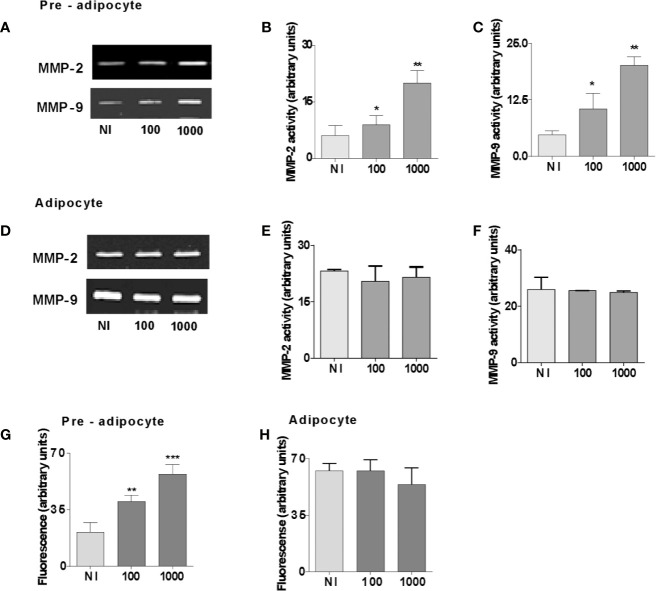
*B. abortus* infection induces MMPs secretion in pre-adipocytes but was unable to modulate MMPs in adipocytes. MMP-2 and MMP-9 activity were measured by gelatin zymography in culture supernatants from *B. abortus*-infected preadipocytes **(A)** and adipocytes **(D)** at 24 h post-infection. **(B, C, E**, and **F)**. Densitometric analysis of results from three independent experiments performed in A and D. For MMP activities, the culture supernatants from B. abortus infected preadipocytes **(G)** and adipocytes **(H)** with a fluorescein-conjugated gelatin substrate that produces highly fluorescent peptides when gelatin is digested. Data are expressed in fluorescence units informed by the fluorometer. Data are given as the mean ± SD measured in triplicate from four individual experiments. Data shown are from a representative experiment of five performed *P < 0.05; **P < 0.01; ***P < 0.001 vs non infected cells (NI).

Cytokines and adipokines have been shown to stimulate MMPs secretion from different cell types (24–30). Additionally, pre-adipocytes and adipocytes can produce MMP-2 and MMP-9 (15). To analyze whether *B. abortus* is capable of modulating their MMP-2 and MMP-9 activity, separated pre-adipocyte and adipocyte cells were infected with *B. abortus* at MOI 100 and 1,000, and after 24 h MMPs activity was evaluated, in culture supernatants, by gelatin zymography. Supernatants, from *B. abortus*-infected pre-adipocyte displayed a significant increase in MMP-2 and MMP-9 gelatinase activity than that of uninfected cells (**Figures 3A–C**). By contrast, in adipocytes, no further increase of the MMP-2 and MMP-9 activity was detected for any MOI of infection (**Figures 3D–F**). The activity of MMPs *in vivo* is counterbalanced by the tissue inhibitor’s action including TIMPs (31). Therefore, the net gelatinase or collagenase activity in a complex sample, such as culture supernatants, depends on the balance between MMP and TIMP activities. This net activity is not revealed by zymography, since MMP-TIMP complexes may dissociate during gel electrophoresis. To assess whether the environment surrounding *Brucella*-infected pre-adipocytes and adipocytes has an increased net gelatinase activity, culture supernatants from these cells were incubated with a non-ﬂuorescent gelatin-ﬂuorescein conjugate, and the ﬂuorescence unmasked as a consequence of gelatin degradation was measured in a ﬂuorometer. Our results indicated that enzymatic activity is increased significantly in *B. abortus*-infected supernatants from pre-adipocytes supernatants but it remained unaltered in supernatants from *B. abortus*-infected adipocytes (**Figures 3G, H**). Therefore, *B. abortus* infection increases the MMP-2 and MMP-9 activity in pre-adipocytes, but no in adipocytes.

### *B. abortus* Lipoproteins Inhibit Adipocyte Differentiation

**Figure 4:**
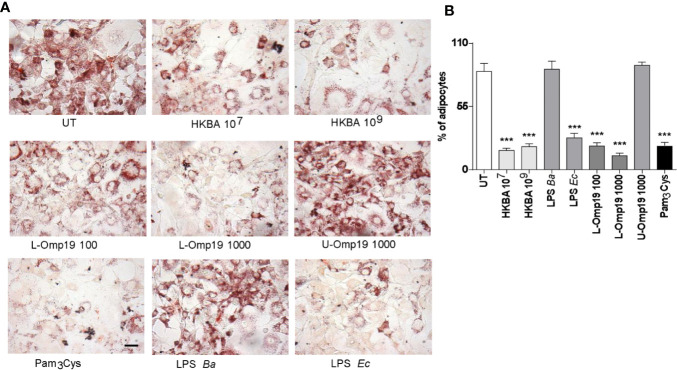
*B. abortus* lipoproteins inhibits adipocyte differentiation. Pre-adipocytes were differentiated in the presence of HKBA (1x106 and 1x109 bacteria/ml) and *E. coli* LPS (LPS *Ec*) (10 ng/ml), *B. abortus* LPS (LPS *Ba*) (1,000 ng/ml), L-Omp19 (10, 100, or 1,000 ng/ml), U-Omp19 (1,000 ng/ml), or Pam3Cys (50 ng/ml) or untreated (UT). The presence of adipocytes was revealed by staining of lipid droplets with Oil Red O **(A)**. Quantification of Oil Red O was determined by cell counts **(B)**. Ten microscopic fields per well in three wells per condition were quantified for each experiment. The percentage of adipocytes was calculated to the untreated cells (UT). Scale bar: 30 µm. Data are given as the mean ± SD measured in triplicate from five individual experiments. Data shown are from a representative experiment of five performed ***P < 0.001 vs untreated cells (UT).

Previously, we have reported that *Brucella* lipoproteins are crucial for many responses induced by *B. abortus* infection (19–21, 32–36). Additionally, adipogenesis inhibition may be a process involving TLR-ligand interactions (37, 38). At first, we evaluated whether viable *B. abortus* was necessary to inhibit adipocyte differentiation. Oil red staining of adipocytes differentiated in the presence of HKBA revealed a markedly reduced adipocyte differentiation compared with the unstimulated cells. We further assessed the *B. abortus* LPS or its lipoproteins involvement on adipocyte differentiation inhibition. To this end, pre-adipocytes were differentiated in the presence of *B. abortus* LPS, or *B. abortus* lipidated outer membrane protein 19 (L-Omp19), used as a model of *Brucella* lipoprotein, and compared against unlipidated (U)-Omp19 (20). LPS from *E. coli* and Pamp_3_Cys were used as positive controls. As shown in **Figure 4**, L-Omp19, but not *B. abortus* LPS, mimicked the inhibition of lipid droplets accumulation induced by *B. abortus* infection. Nonetheless, the U-Omp19 was not capable to inhibit lipid droplets accumulation (**Figure 4**). Together these results indicate that the lipoproteins (L-Omp19) from *B. abortus* but not its LPS participates in the inhibition of adipogenesis among infected pre-adipocytes.

### *B. abortus*-Infected Macrophages Modulate Adipocyte Differentiation

**Figure 5:**
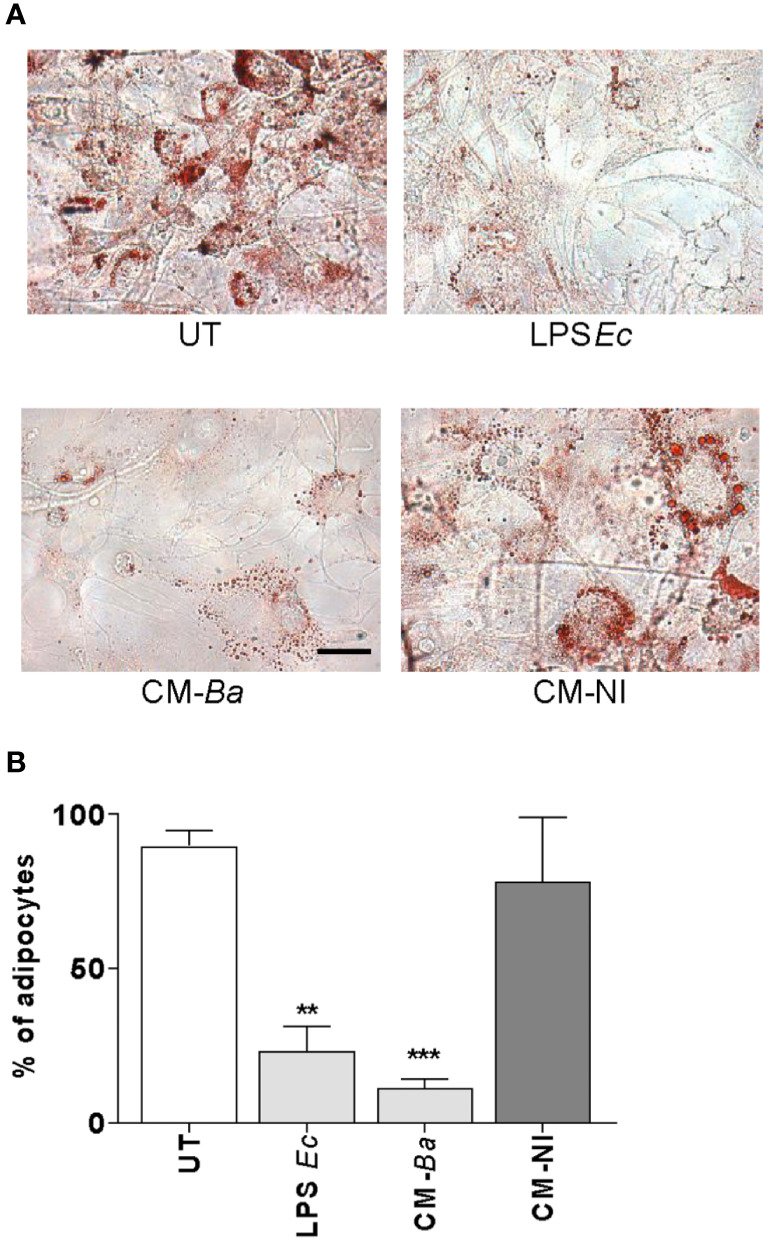
*B. abortus*-infected macrophages modulate adipocyte differentiation. Pre-adipocytes were differentiated in the presence of culture supernatants from *B. abortus* infected macrophages at MOI 100 (CM-Ba) or culture supernatants from non-infected macrophages as control (CM-NI). The presence of adipocytes was revealed by staining of lipid droplets with Oil Red O **(A)**. Quantification of Oil Red O was determined by cell counts **(B)**. LPS from *E. coli* (LPS *Ec*) was used as a positive control. Ten microscopic fields per well in three wells per condition were quantified for each experiment. The percentage of adipocytes was calculated with respect to untreated cells (UT). Scale bar: 30 µm. Data are given as the mean ± SD measured in triplicate from five individual experiments. Data shown are from a representative experiment of five performed. **P < 0.01; ***P < 0.001 vs untreated cells (UT).

Macrophages constitute the main host cell for *B. abortus* replication (39). We hypothesized that *B. abortus*-infected macrophages release soluble mediators that may affect adipocyte differentiation. For this goal, the adipocyte differentiation process was studied in the presence of conditioned media from *B. abortus*-infected macrophages, or uninfected macrophages as control. As shown in **Figure 5**, the adipocyte differentiation was significantly reduced by conditioned media from *B. abortus*-infected macrophages but it was not modified by conditioned media from uninfected macrophages (**Figure 5**).

**Figure 6:**
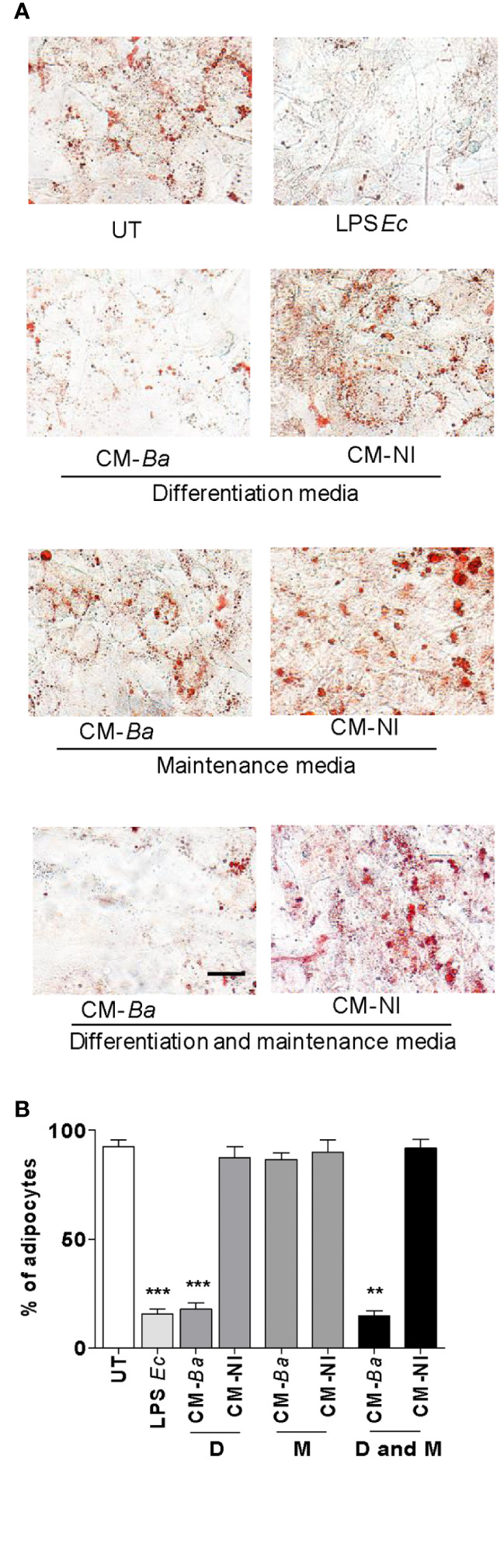
*B. abortus*-infected macrophages modulate early adipocyte differentiation. Pre-adipocytes were differentiated in the presence of culture supernatants from *B. abortus*—infected macrophages at MOI 100 (CM-Ba) or culture supernatants from non-infected macrophages as control (CM-NI). Supernatants were added during the early differentiation process (differentiation media, D), later differentiation process (maintenance media, M) or during all differentiation process (differentiation and maintenance media, D and M). The presence of adipocytes was revealed by staining of lipid droplets with Oil Red O **(A)**. Quantification of Oil Red O was determined by cell counts **(B)**. LPS from *E. coli* (LPS *Ec*) was used as a positive control. Ten microscopic fields per well in three wells per condition were quantified for each experiment. The percentage of adipocytes was calculated to the untreated cells (UT). Scale bar: 30 µm. Data are given as the mean ± SD from three individual experiments. **P < 0.01; ***P < 0.001 vs untreated cells (UT).

To determine whether conditioned media from *B. abortus*-infected macrophages affects at early and/or late stage of the adipocyte differentiation process, we added the conditioned media from *B. abortus*-infected macrophages during the incubation with adipocyte differentiation medium or, during the cultivation with maintenance medium. As a control, conditioned media from uninfected macrophages was included. The adipocyte differentiation was inhibited when conditioned media from *B. abortus*-infected macrophages was added during the culture of cells with adipocyte differentiation media indicating that suppresses the pre-adipocyte differentiation at an early stage while the addition of conditioned medium during the cultivation with maintenance medium did not affect adipocyte differentiation (**Figure 6**). Together, these results indicate that soluble factors released from *B. abortus*- infected macrophages inhibit adipogenesis at an early state.

### *B. abortus*-Infected Macrophages Inhibit Adipocyte Differentiation *via* a Mechanism Dependent on TNF-α

**Figure 7:**
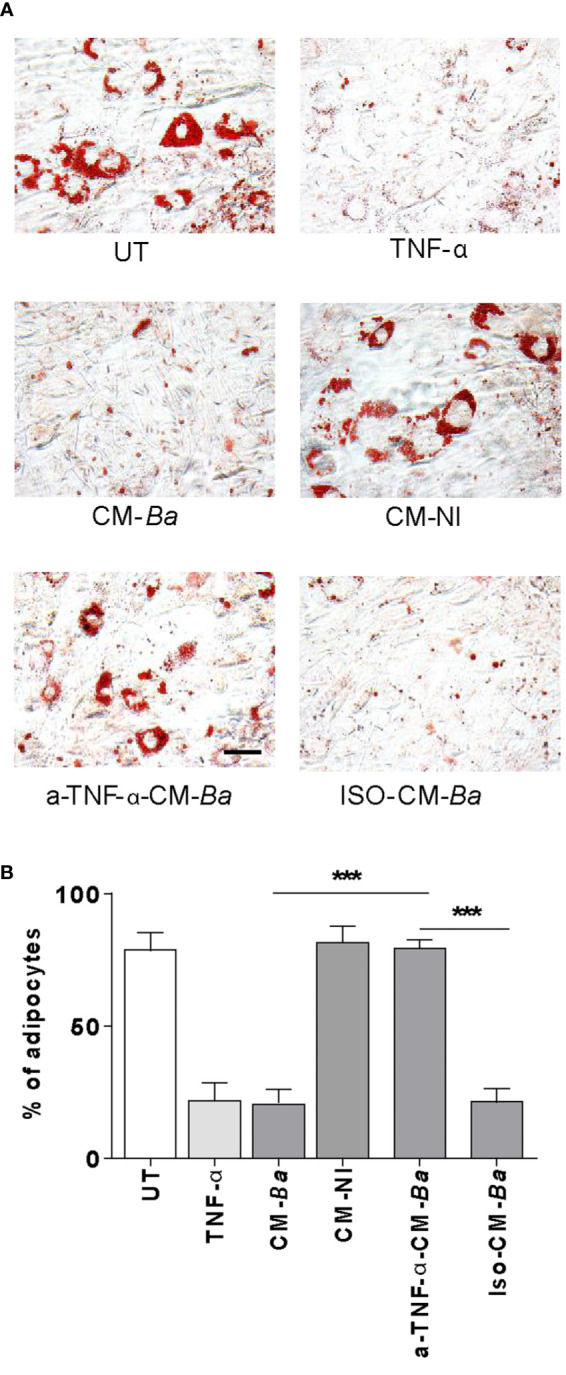
*B. abortus*-infected macrophages inhibit adipocyte differentiation *via* a mechanism dependent on TNF-α. Pre-adipocytes were differentiated in the presence of culture supernatants from *B. abortus*-infected macrophages at MOI 100 (CM-Ba) or culture supernatants from non-infected macrophages as control (CM-NI). Supernatants were incubated with an anti-TNF-α neutralizing antibody (a-TNF-α-CM-Ba) or isotype control (ISO-CM-Ba). TNF-α (1 ng/ml) was used as a positive control. The presence of adipocytes was revealed by staining of lipid droplets with Oil Red O **(A)**. Quantification of Oil Red O was determined by cell counts **(B)**. Ten microscopic fields per well in three wells per condition were quantified for each experiment. The percentage of adipocytes was calculated to the untreated cells (UT). Scale bar: 30 µm. Data are given as the mean ± SD from three individual experiments. ***P < 0.001.

TNF-α is a proinflammatory cytokine able to downregulate the adipocyte differentiation (40). Previously, we have reported that *B. abortus*-infected macrophages secrete TNF-α (21, 32). To test whether TNF-α released from *B. abortus*-infected macrophages inhibits adipogenesis, we performed experiments with culture supernatants preincubated with a TNF-α neutralizing antibodies. As shown in **Figure 7**, the inhibitory effect of *B. abortus*-infected macrophages on adipocyte differentiation was inhibited by the treatment with TNF-α neutralizing antibody. Also, the isotype control does not affect. Thus, we concluded that TNF-α plays a key role in the inhibition of adipogenesis induced by macrophages-infected with *B. abortus*.

## Discussion

The role of adipose tissue in the pathogenesis of infectious diseases has increased attention in recent years (41–45). Adipose tissue constitutes a nutritionally rich organ for the survival of pathogens such as *Mycobacterium tuberculosis*, *Trypanosoma cruzi*, HIV, among others (9, 46, 47). Cell types composing adipose tissue include, fibroblasts, smooth muscle, endothelial, and immune cells. In the setting of infectious or non-infectious diseases like diabetes or obesity, adipose-resident immune cells are derived to a proinflammatory phenotype due to a proinflammatory microenvironment. However, adipocytes are the most abundant cell type (48).

Cell types composing adipose tissue include, fibroblasts, smooth muscle, endothelial and immune cells. In the setting of infectious or non-infectious diseases like diabetes or obesity, adipose-resident immune cells are derived to a proinflammatory phenotype due to a proinflammatory microenvironment.

In brucellosis, the tissue and cell type in which the bacteria persist during chronic infection remains unknown. Previous findings have reported the capacity of *B. abortus* for intracellular replication in several cell types (17, 21, 49). In particular, the *Brucella* capability to localize intracellularly in adipocytes and its precursors was recently revealed for *B. canis* (13, 17). Here, we show that *B. abortus* infects and replicates in both adipocyte and even with higher efficiency, in pre-adipocyte.

An impairment in adipogenesis process may involve an adipokine misbalance induced by *B. abortus* infection. Adiponectin and resistin have been associated with adipogenesis (50, 51). Leptin acts largely on the hypothalamus, informing the nutritional status of the adipocytes and controlling adipokines and the endocrine role of adipose tissues and food intake, thus regulating energy balance (23). Additionally, leptin could significantly reduce lipid accumulation during the adipocyte differentiation process, indicating an inhibitory effect on adipogenesis. The loss of expression of adipokines and the acquisition of an inflammatory phenotype may also involve the secretion of IL-1β, TNF-α, and IL-6 (52). Here, we have demonstrated an increased secretion of adipokines such as IL-6, but not IL-1β and TNF-α, among infected pre-adipocytes and adipocytes after *B. abortus* infection, being higher among the former. Likewise, pre-adipocytes depict a higher number of intracellular bacteria than adipocyte cells. Considering that IL-6 could be able to inhibit the anti-inflammatory adipokines adiponectin and resistin in an autocrine and paracrine manner (53), we have measured their transcriptional level after *B. abortus* infection. For both, the mRNA level appeared significantly diminished after infection. However, although IL-6 also induces leptin production (54, 55), leptin levels were not significantly altered after infection with *Brucella abortus*. Several transcription regulators, such as PPAR-γ, C/EBP-α, and C/EBP-β are well-known to mediate adipogenesis. The main role of PPAR-γ in adipogenesis during infection has been revealed using *in vivo* models of infectious diseases (41, 44). Moreover, the expression level of the critical adipogenic transcription factor PPAR-γ was downregulated after *B. abortus* infection, thus affecting its role in the maintenance of mature adipocyte phenotype (56, 57). C/EBPα is expressed in the late phase of adipocyte differentiation. In contrast, C/EBPβ is expressed very early during adipogenesis and is also required to sustain the expression of PPAR-γ and C/EBPα. After the treatment of preadipocytes with inducers of differentiation, a rapid and transient increase in transcription and expression of C/EBPβ occurs and then decrees during the differentiation process (14). As we expected, *B. abortus* infection also inhibited C/EBPα transcription (58). On the contrary, *B. abortus* infection increases the transcription of C/EBPβ. These contradictory results could be explained, at least in part, by the fact that the transcriptional activity of C/EBPβ is regulated by several posttranscriptional modifications (59), including acetylation (60, 61), phosphorylation, and polyubiquitination (62). Additionally, it could be also speculated that overexpression of C/EBPβ may be a compensatory mechanism in response to C/EBPα reduction as was described for brown adipocyte differentiation (63). Further studies are needed to define the mechanism involved during *B. abortus* infection and the significance of this regulation.

We have observed that *B. abortus* infection of pre-adipocytes may also alter the extracellular matrix by inducing MMP-2 and MMP-9 secretion. Despite this, finding appears to be opposed to previous studies that reported that these MMPs are involved in extracellular matrix remodeling during adipocyte differentiation (15, 64, 65), two plausible explanations should be considered. First, it is known that other MMPs such as MMP-3 could have the opposite effects and down modulate the adipogenic differentiation (66, 67). Second, the MMPs action is controlled by regulators such as tissue inhibitor of metalloproteinases (TIMPs) as well as members of protease family of ADAMs (68). *In vivo*, TIMPs counterbalanced the activity of MMPs, and these complexes are dissociated during gelating zymography procedure. However, in general, in inflammatory conditions, TIMPs do not increase in the same degree as MMPs do, thus increasing the MMP/TIMP ratio (4, 69). Accordingly, supernatants from *B. abortus*-infected pre-adipocytes produced gelatin cleavage when evaluated under native conditions in the fluid phase, with MMP-TIMP complex are not dissociated as occurs during gel electrophoresis. This indicates that other mechanisms are involved during adipogenesis inhibition by *B. abortus* infection.

Adipogenesis inhibition may be a process involving TLR-ligand interactions (37, 38). Here, we have demonstrated that *B. abortus*-mediated inhibition of the adipocyte differentiation was independent of the bacterial viability, thus insinuating a potential role elicited by a structural bacterial component. As in our previous studies, the bacteria lipoprotein L-Omp19 plays again a critical role in this sense but not the LPS (20). Such lipid moiety but not its non-lipidated counterpart U-Omp19, was able to mimic adipogenesis inhibition induced by *B. abortus* infection. The effect was elicited by the lipid moiety, which is likely shared by all bacterial lipoproteins. The genome of *B. abortus* codifies no less than 80 putative lipoproteins (70). This indicates that lipoproteins present in *Brucella* could be sufficient to modulate adipocyte physiology.

In adipose tissue, immune cells including macrophages and T cells are the main responsible for inflammatory cytokine production (71, 72). In particular, macrophages participate in adipose tissue dysfunction and reduced adipogenesis (73, 74). *Brucella* infection is accompanied by the infiltration of inflammatory cells, and the macrophages represent the main replication niche for these bacteria (75). Even though the role of macrophage polarization in the pathogenesis of *Brucella* species is poorly described until now (76, 77). Here, we have demonstrated that proinflammatory cytokines -such as TNF-α- secreted by macrophages in response to *B. abortus* infection exert an inhibitory effect on adipogenic differentiation (52). However, we cannot rule out that other cytokines are also present in the conditioned media and contribute to these observations.

Overall, these results suggest a possible scenario in which *B. abortus* infection *via* its lipoproteins modulates adipogenesis process and soluble mediators secreted by *B. abortus*—infected macrophages may contribute to this phenomenon, as a mechanism in which TNF-α is involved.

In conclusion, our results suggest that adipogenesis process could be altered directly after exposure—even without effective multiplication—of the adipocytes and their precursors to *Brucella*, or their lipoproteins. Furthermore, this same process could be indirectly altered, thanks to soluble mediators such as TNF-α released by macrophages infected by the same bacteria.

These early studies using murine cell lines provide clues regarding potential mechanisms involved during the interaction of *B. abortus* with adipocytes. Further studies using primary human adipocytes, human adipose tissue explants, and the *in vivo* murine model will be needed to confirm whether the responses described here have a role in the chronic inflammation and chronicity of the infection.

## Data Availability Statement

The raw data supporting the conclusions of this article will be made available by the authors, without undue reservation.

## Author Contributions

AP performed the experiments. All authors analyzed the data. MD and JQ wrote the article. MD and JQ designed the experiments, revised the article, and obtained research funding. GG performed a critical read of the manuscript. All authors contributed to the article and approved the submitted version.

## Funding

This work was supported by grants from Agencia Nacional of Promoción Científica y Tecnológica (ANPCYT, Argentina), PICT 2014-1111, PICT 2015-0316, PICT 2017-2859 to MD and PICT-2015-1921 to JQ. Funding agencies had no role in study design, data collection and analysis, decision to publish, or preparation of the manuscript.

## Conflict of Interest

The authors declare that the research was conducted in the absence of any commercial or financial relationships that could be construed as a potential conflict of interest.
